# Accumulation of As, Cd, and Pb in Sixteen Wheat Cultivars Grown in Contaminated Soils and Associated Health Risk Assessment

**DOI:** 10.3390/ijerph15112601

**Published:** 2018-11-21

**Authors:** Guanghui Guo, Mei Lei, Yanwen Wang, Bo Song, Jun Yang

**Affiliations:** 1 Institute of Geographic Sciences and Natural Resources Research, Beijing 100101, China; guogh@igsnrr.ac.cn (G.G.); wangyw@igsnrr.ac.cn (Y.W.); yangj@igsnrr.ac.cn (J.Y.); 2 College of Environmental Science and Engineering, Guilin University of Technology, Guilin 541004, China; songbo@glut.edu.cn

**Keywords:** bioaccumulation factors, health risk, target hazard quotient, *Triticum aestivum* L.

## Abstract

This study investigated the accumulation of As, Cd, and Pb in 16 wheat cultivars and the associated health risks for the inhabitants of Jiyuan, China. The results indicated that the concentrations of As, Cd, and Pb decreased in the order of root > leaf > stem > grain. The concentrations of As, Cd, and Pb in wheat grains varied from 0.13 for Pingan8 to 0.34 mg kg^−1^ for Zhengmai7698, 0.10 for Luomai26 to 0.25 mg kg^−1^ for Zhengmai7698, and 0.12 for Zhoumai207 to 0.42 mg kg^−1^ for Zhengmai379, respectively. There were significant differences in the bioaccumulation factors of As, Cd, and Pb among the 16 wheat cultivars. Cd was more readily accumulated to higher levels than As and Pb in wheat. The Target Hazard Quotients (THQs) of Cd and Pb in the grains from 16 wheat cultivars were below 1, while As THQ exceeded 1. The lowest detrimental human health effects via wheat consumption were found in cultivar AY58 among the 16 wheat cultivars, with total THQs (TTHQs) of 1.82 for children and 1.60 for adults, suggesting that children absorb more heavy metals than adults and they are more vulnerable to the adverse effects of these metals.

## 1. Introduction

Soil contamination with heavy metals has become a serious environmental problem in recent years due to the rapid economic development and increased human activities [1]. Heavy metals cannot be degraded naturally and therefore persist in soils for a long period of time [2]. It was reported that approximately one fifth of the farmland in China is contaminated with arsenic (As), cadmium (Cd), and lead (Pb), which has generated concerns on the crop productivity and food safety [3,4,5]. Food consumption had been identified as the major exposure pathway to As, Cd, and Pb, compared with other pathways of exposure, such as inhalation and dermal contact. Furthermore, As, Cd, and Pb can cause detrimental effects on humans even at very low concentrations when exposure occurs over a long time period [6]. As, Pb, and Cd are classified on the priority list of hazardous substances by the United States Environmental Protection Agency (USEPA) [7]. Chronic As exposure can cause serious health effects, including skin, bladder, and lung cancer, neuropathy, gastrointestinal diseases, cardiovascular diseases, and other ailments [8,9]. Cd can adversely affect the function of the immune system and cause several diseases, like osteoporosis, nonhypertrophic emphysema, irreversible renal tubular injury, and other diseases [10,11,12]. Pb exposure can influence the neurologic systems, kidneys, and blood circulation, especially in children, infants, and fetuses [13,14]. Therefore, it is necessary to decrease the accumulation of As, Cd, and Pb in crops to ensure food safety.

Wheat (*Triticum aestivum* L.) is the third most important cereal worldwide after the rice and maize. Wheat is the dominant staple food and one of the main dietary components for many Chinese people, especially in North China [15]. Wheat consumption accounted for a large proportion of the agricultural produces, and wheat was considered as an important source of minerals [16]. However, As and heavy metals can be readily taken up by wheat roots, and can be accumulated at high levels in wheat grains, even at low levels in the soils [17]. Many studies investigated the regularity of migration and accumulation of As and heavy metals between soil and crops. Ren et al. investigated the effects of Zn and Cd on the growth of wheat by a pot experiment [18]. The stress of Cd, Pb, As, Hg, and Cr on wheat yields and ingredients in the farmland were studied by Nie et al. [19]. Wheat consumption is considered to be one of the major sources of As and heavy metals intake for humans, which can affect human health. Until now, the research on As, Cd, and Pb has mainly focused on the physiological mechanisms of pollution remediation and risk assessment of pollution. In addition, these studies have mainly focused on the behavior of a single metal in soil–plant systems, but there is little information available on the selection of cultivars with a low accumulation of multiple heavy metals. It was noticeable that many studies on As, Cd, and Pb accumulation were conducted under pot experiment or hydroponic conditions; however, field investigations are important to examine the differences in multiple heavy metals and As accumulation among wheat cultivars. Accordingly, a selection of wheat species or cultivars with low As and heavy metal accumulation, rather than leaving the farmland fallow, is potentially a suitable method to reduce the adverse health effects of As and heavy metals. This will provide important information for the selection of cultivars with low heavy metal concentration to reduce food chain contamination and potential human health risks. 

Therefore, the aim of this study was to (1) investigate the As, Cd, and Pb concentrations in different tissues of wheat cultivars and compare the bioaccumulation factors; and (2) assess the human health risks of individual and total heavy metals from different wheat cultivars.

## 2. Materials and Methods

### 2.1. Experimental Site

The field experiment site was located on farmland with heavy metal contamination in Tangshi village, Jiyuan, Henan Province, China (112°31′21.319′′ E, 35°08′24.918′′ N). The study site is in a warm tropical climate zone, with an average annual temperature of 14.9 °C, average annual rainfall of 600.3 mm, and a frost-free duration of 233 days per year. The soils in the study area belonged to Ustic Cambo soils according to the Chinese Soil Taxonomy [20]. A large agricultural area is used mainly as farmland with a winter wheat-summer corn rotation as the dominant planting system.

**Figure 1:**
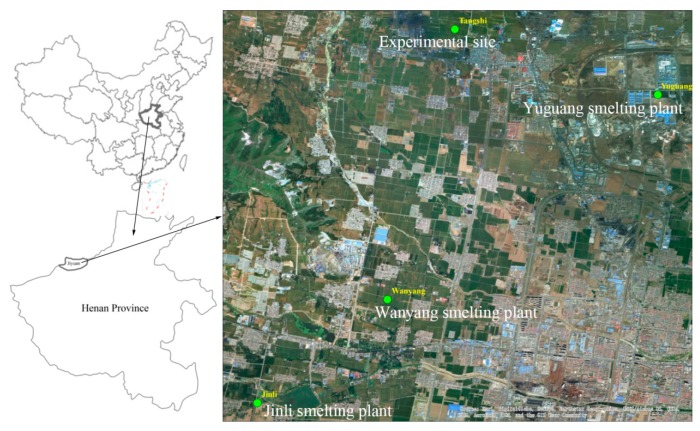
The location of the experimental site and smelting plants.

In recent years, Jiyuan has become the largest Pb smelting and processing base in China. In 2017, it produced 112,100 t of Pb, which accounted for 73.97% of the total Pb produced in Henan Province. Three major Pb smelting plants (Yuguang, Jinli, and Wanyang plants) are located in Jiyuan (Figure 1). These smelting plants have contributed to local economic development, but have also resulted in the emission of As, Cd, and Pb into the air, water, and soils from the smelting activity [21,22]. Despite that, some farmlands in the area around Jiyuan are contaminated with As, Cd, and Pb, and these farmlands continue to produce crops, such as wheat and maize. These foodstuffs are eaten by the farmer or taken to the market for sale to local residents.

### 2.2. Wheat Cultivars

A total of 16 winter wheat cultivars, including Bainong58, Zhoumai207, Luomai26, Zhengmai7698, Xuke718, Zhengmai366, AY58, Zhoumai18, Zhengmai379, Zongmai583, Xinong979, Zhengmai0856, Zhoumai0856, Fengdehou, Pingan8, and Fanmai8, were used in this study. The seeds of 16 wheat cultivars used in the experiment were collected from Henan academy of agricultural sciences, Henan Province, China. Four wheat cultivars, including Zhengmai7698, Zhengmai366, Zhengmai379, and Zhengmai0856, were the newly developed cultivars. The other 12 wheat cultivars, including Bainong58, Zhoumai207, Luomai26, Xuke718, AY58, Zhoumai18, Zongmai583, Xinong979, Zhoumai0856, Fengdehou, Pingan8, and Fanmai8, were commercial wheat cultivars, which were widely used in traditional farming systems in Henan Province.

### 2.3. Field Experiments

The open field experiment was a randomized complete block split-plot design (*n* = 48) with 3 replicates for each of the 16 wheat cultivars. The area of each plot was 50 m^2^ (25 m long and 2 m wide). Nitrogen (N), phosphorus (P), and potassium (K) compound fertilizer was used as a basal fertilizer (N:P:K = 25:12:8) prior to sowing at a rate of 750 kg ha^−1^. At the stem jointing and booting stages, 0.5% urea (225 kg ha^−a^) was used for topdressing. Wheat seeds (1.5 kg per plot) were sown on 28 September 2016, and then harvested on 3 June 2017. Irrigation was applied three times during the experiment. The field was managed according to conventional operating practices.

### 2.4. Sample Collection

Prior to sowing, 48 composite soil samples (500 g) composed of 5 separate subsamples were collected from the surface layer (0–20 cm) of the experimental farmland. 

At harvest, 5 subsamples of the wheat plants from each plot were mixed and designated as a composite sample. Meanwhile, the rhizosphere soil of each wheat sample was separated from the roots. At last, 48 rhizosphere soils samples and 48 wheat samples were collected from the experimental spots. The wheat and soil samples were stored in polyethylene bags at a constant temperature of 4 °C and then transported to the laboratory.

### 2.5. Sample Preparation and Chemical Analysis

The soil samples were air-dried, and then ground with a stainless-steel grinder (MM400, Retsch, Germany) to pass through a 0.15-mm polyethylene sieve. Soil pH was measured in 1:2.5 (*w*:*v*) of soil:water suspension using a glass electrode. Soil organic matter was determined by oxidation with potassium dichromate and colorimetric determination, which consisted in oxidizing an aliquot of soil (0.5 g) with a solution of K_2_Cr_2_O_7_ + H_2_SO_4_ at 160 °C. The excess of dichromate is titrated with 0.25 mol/L FeSO_4_. Cation exchange capacity (CEC) was assessed via a 1 mol/L NH_4_Oac (AR, Xilong, China) leaching method (pH = 7). Total N, total P, total K, alkali hydrolysable N, rapidly-available P, and rapidly-available K were analyzed using the routine analytical methods of agricultural chemicals and soils [23]. The total N, K, and P in the soils were determined by kjeldahl nitrogen distillation, Mo-Sb colorimetric method, and atomic absorption spectrometry, respectively. Soil samples (0.5 g) were digested with nitric acid (HNO_3_) and hydrogen peroxide (H_2_O_2_) using Method 3050B of the USEPA [24], and the digest solution was diluted to 50 mL with ultrapure water.

The whole plants were washed thoroughly with tap water and then rinsed with deionized water several times to remove dust and impurities. The plants were divided into root, stem, leaf, and grain. These samples were then oven-dried at 50–60 °C to achieve a constant weight. The dried samples were ground in a stainless-steel grinder chamber (MM400, Retsch, Germany) and passed through a 1-mm polyethylene sieve before digestion. Powdered sample (1.0 g) was digested in a mixture of 5 mLnitric acid (HNO_3_) and 2 mL perchloric acid (HClO_4_) solution, and then heated with an electric hot plate (up to 150 °C) to obtain 0.5 mL of colorless solution [25]. The digestion solution was transferred to a flask and diluted with 50 mL with ultrapure water.

The As concentrations in the digested solutions were determined using an atomic fluorescence spectrometer (KCHG AFS-9800 Beijing Haiguang Instrument, Beijing, China). The Cd and Pb concentrations in the digested solutions were determined by inductively coupled plasma mass spectrometer (ICP-MS, ELAN DRC-e, Perkin Elmer, Waltham, MA, USA).

### 2.6. Quality Control and Quality Assurance

Quality assurance procedures and precautions were carried out to ensure the accuracy and precision of the results. Certified reference materials for soil (GBW07406) and wheat (GBW10035) were obtained from the National Research Center for Standards of China. Duplicate samples and blank samples were included in each batch of analyses for quality control purposes. All samples were analyzed in triplicate and the results were accepted when the relative standard deviation was within 5%. The method detection limit was 0.03 mg/kg for As. The method detection limit was 0.3 ng/kg for Cd and Pb. The recoveries (mean ± SD) of As, Cd, and Pb were 95.33 ± 3.2%, 98.4 ± 5.6%, and 96.5 ± 6.4%, respectively.

### 2.7. Data Analysis

#### 2.7.1. Bioaccumulation Factors (BCFs)

The BCFrs, BCFss, BCFls, and BCFgs were calculated to evaluate the ability of the plant to accumulate As, Cd, and Pb as follows:

(1) $${\mathrm{BCF}}_{\mathrm{rs}}=\frac{C_{root}}{C_{soil}}$$

(2) $${\mathrm{BCF}}_{\mathrm{ss}}=\frac{C_{stem}}{C_{soil}}$$

(3) $${\mathrm{BCF}}_{\mathrm{ls}}=\frac{C_{leaf}}{C_{soil}}$$

(4) $${\mathrm{BCF}}_{\mathrm{gs}}=\frac{C_{grain}}{C_{soil}}$$

where *C_root_*, *C_stem_*, *C_leaf_*, and *C_grain_* were the average concentrations of As, Cd, and Pb in the wheat roots, stems, leaves, and grains, respectively. *C_soil_* was the average concentration of As, Cd, and Pb in the rhizosphere soil.

#### 2.7.2. Health Risk Assessment

The potential health risks of As, Cd, and Pb via wheat consumption were assessed based on the target hazard quotient (THQ) method developed by the USEPA [26,27]. The THQ was calculated by the following equation:

(5) $$\mathrm{THQ}=\frac{\mathrm{EF}\times \mathrm{ED}\times \mathrm{DI}\times C}{\mathrm{RfD}\times \mathrm{BW}\times \mathrm{AT}}\times 10^{-3}$$

where EF is the exposure frequency (350 d y^−1^), ED is the exposure duration (30 years for adults, 6 years for children), daily intake (DI) is the average daily wheat consumption (159.9 g per person per·day (g p^−1^·d^−1^) for adults and 94.47 g p^−1^·d^−1^ for children) [28,29], C is the concentrations of heavy metal(loid)s in the wheat grains (mg kg^−1^), BW is the average body weight (63.9 kg for adults and 32.7 kg for children), AT is the average exposure time (AT = ED × 365 d y^−1^ for non-carcinogenic risk), and RfD is the reference dose for As, Cd, and Pb (As: 0.05 mg kg^−1^·d^−1^; Cd: 0.001 mg kg^−1^·d^−1^; Pb: 0.0035 mg kg^−1^·d^−1^) [30,31].

The total THQ (TTHQ) of heavy metal(loid)s via wheat consumption was calculated as the sum of the THQs, as shown in the equation below:

(6) $$\mathrm{TTHQ}=\overset{n}{\underset{i=1}{\sum }}{\mathrm{THQ}}_{i}$$

Detrimental health effects are assumed to be unlikely if THQ ≤ 1 or TTHQ ≤ 1, but potential effects are assumed to be possible if THQ > 1 or TTHQ > 1 [32].

#### 2.7.3. Statistical Analysis

Statistical analyses were performed using SPSS 19.0 software (IBM Corporation, New York, NY, USA). The non-parametric test (Kolmogorov-Smirnov) was performed to test the significance of As, Cd, and Pb concentrations in wheat grains. Spearman correlation was used to compare the concentrations of As, Cd, and Pb in the wheat grains with the soil parameters. The figures were produced using Origin 9.0 software (Origin Lab Corporation, Northampton, MA, USA).

## 3. Results and Discussion

### 3.1. Soil Properties

The physical and chemical properties of the surface soils (0–20 cm) in the experimental site are presented in Table 1. The soil pH was 7.23 (weakly alkaline). The soil had an average organic matter (OM) content of 16.8 g kg^−1^, with the range from 15.5 to 18.10 g kg^−1^. The total N, P, and K concentrations were 0.139%, 0.069%, and 1.97%, respectively. The concentrations of As, Cd, Pb, Cr, Cu, Ni, and Zn in the soils ranged from 14.57 to 22.40, 1.69 to 2.35, 142.63 to 210.23, 75.31 to 90.47, 22.60 to 30.67, 23.79 to 30.87, and 76.90 to 102.88 mg kg^−1^, respectively, with mean values of 12.31, 2.06 177.37, 80.81, 25.42, 26.23, and 87.15 mg kg^−1^, respectively. The soil As, Cr, Cu, Ni, and Zn concentrations did not exceed the corresponding standard values (GB15618-2018) [33], while the Cd and Pb concentrations in the soils were 6.86 and 1.48 times higher than the corresponding standard values. These results indicated that the soils in this study were contaminated with Cd and Pb.

Compared with the results by Zhu et al. [34], who found that Pb concentration in soils near the smelting plants in Jiyuan was 426.07 mg/kg, lower Pb concentrations were found in this study. Additionally, the results reported by Qu et al. [35] showed higher As, Cd, and Pb concentrations in the soils from Shibin Village in Jiyuan City than the concentrations of metals in this study. Compared with the Cu, Zn, Pb, and Cd concentrations in the soils from Dongdagou and Xidagou (Gansu Province, China) [36], the soils in this study showed lower metal levels. Similar results were reported by Liu et al. [37], who observed that the concentration of Cd in soils from Xuzhou, China was 2.57 mg/kg.

### 3.2. Heavy Metal Concentrations in Wheat Tissues

The concentrations of As, Cd, and Pb in the grains, stems, leaves, and roots of the 16 wheat cultivars are shown in Table 2. Wide variations in the As, Cd, and Pb concentrations in different wheat tissues were found among the 16 wheat cultivars. The highest As, Cd, and Pb concentrations in the wheat grains were 9.29, 2.5, and 3.5 times higher, respectively, than the lowest concentrations. The highest As, Cd, and Pb concentrations in the stems were 4.50, 7.93, and 4.26 times higher, respectively, when compared with the corresponding lowest concentrations. The concentrations of As, Cd, and Pb in the wheat leaves ranged from 1.44 for Bainong58 to 3.31 for Luomai26, 0.81 for Zhoumai0856 to 1.81 for AY58, and 3.81 for Zhengmai0856 to 7.87 for Bainong58 mg kg^−1^, respectively, with differences of 2.29-, 2.23-, and 2.07-times, respectively, between the maximum and minimum values. The As, Cd, and Pb concentrations in the roots varied by approximately 2.39-, 2.01-, and 2.42-fold, respectively, between the highest concentrations and the lowest concentrations, with the range from 4.64 for Fengdehou to 11.12 for Zhoumai18, from 2.23 for Bainong58 to 4.49 for Zhoumai207, and from 41.33 for AY58 to 99.91 mg kg^−1^ for Zhoumai207, respectively. 

Regardless of the wheat cultivars, the As, Cd, and Pb concentrations for different parts of wheat decreased markedly in the order of roots > leaves > stems > grains (Table 3), indicating that the roots act as a barrier for metal accumulation or translocation, and then protect the edible parts from As, Cd, and Pb contamination. As seen in Table 3, the concentrations of Pb and Cd in the wheat grains were significantly lower than those in any other tissues (*p* < 0.05). The concentrations of Pb in stems, leaves, and roots were 7.91, 20.54, and 290.21 times higher, respectively, than those in grains. The Cd concentrations in stems, leaves, and roots were 3.83, 6.44, and 17.22 times higher, respectively, than those in grains. The concentrations of As in grains and stems were significantly lower than those in other tissues (*p* < 0.05), but no significant differences were found between As concentrations in grains and stems (*p* > 0.05). These results were similar to those reported by Wang et al. [38] and Liu et al. [39], who observed that heavy metals in wheat tissues decreased in the following order: root > leaf > stem > grain. 

### 3.3. Comparison of As, Cd, and Pb Concentrations in Wheat Grains with the Maximum Permitted Concentration (MPC)(GB2762-2017 China)

**Figure 2:**
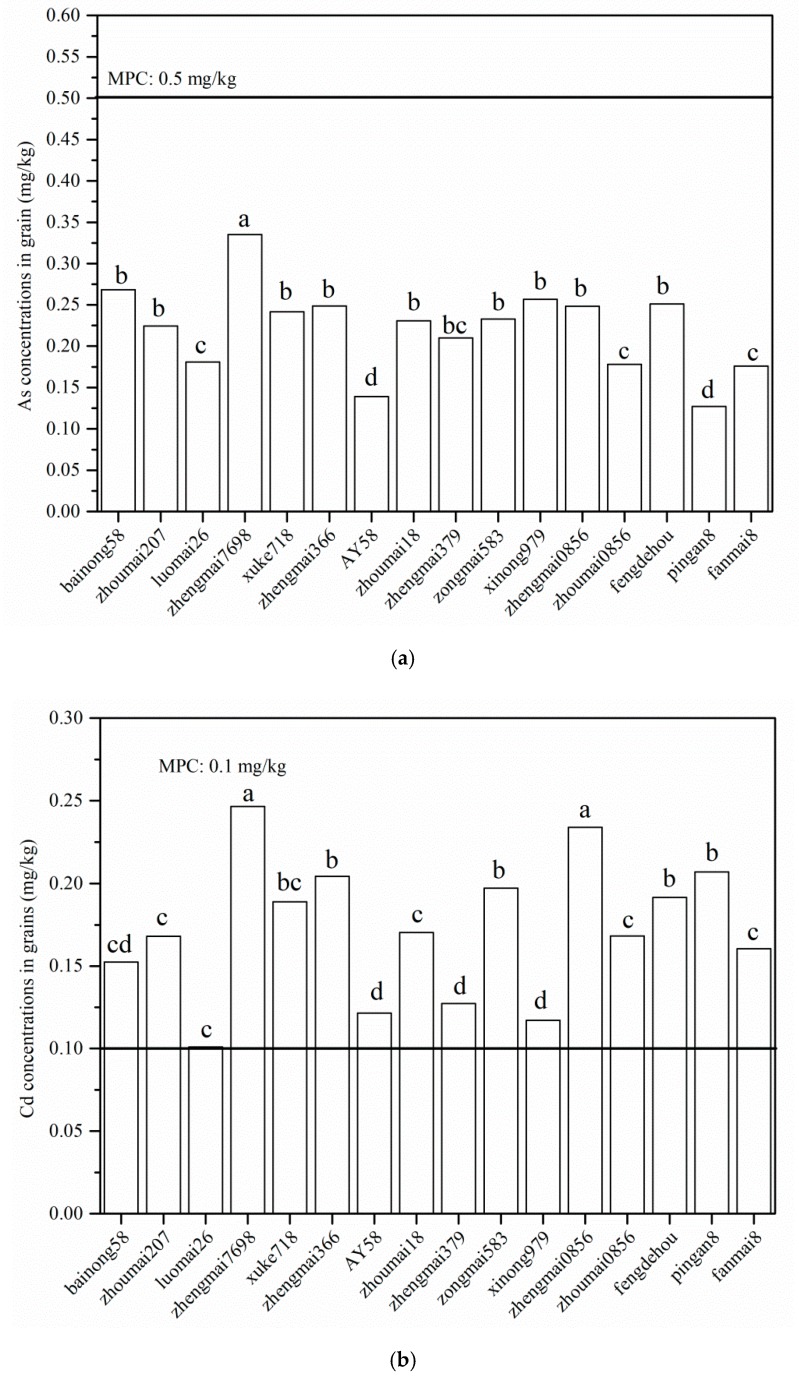

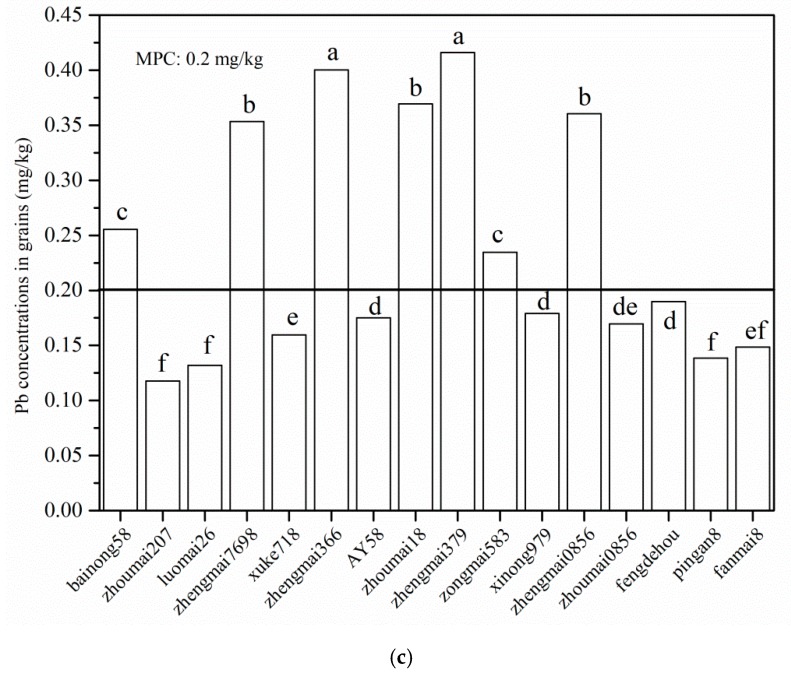
Concentrations of As (**a**), Cd (**b**), and Pb (**c**) in the grains of 16 wheat cultivars. Different letters indicate significant differences in mean values at *p* < 0.05.

As, Cd, and Pb concentrations in grains should be a high priority because of the importance of food safety. The concentrations of As, Cd, and Pb in wheat grains for 16 wheat cultivars are presented in Figure 2, and then the concentrations of As, Cd, and Pb in wheat grains were compared with the MPC values (GB2762-2017) recommended by the Ministry of Health [40].

The concentrations of As in wheat grains varied from 0.13 mg kg^−1^ for Pingan8 to 0.34 mg kg^−1^ for Zhengmai379 (Figure 2a). The As concentration in Pingan8 was significantly lower than that in other wheat cultivars (*p* < 0.05), but there was no significant difference in As concentration between Pingan8 and AY58 (*p* > 0.05). The grain As concentrations for the 16 wheat cultivars were much lower than the MPC value of 0.5 mg kg^−1^. Similar results were also reported by Xing et al. [41], who found that none of the As concentrations in the grains of 25 wheat cultivars exceeded the MPC value in Jiyuan City.

The grains’ Cd concentration ranged from 0.10 mg kg^−1^ for Luomai26 to 0.25 mg kg^−1^ for Zhengmai7698. The Cd concentration in the grains of Zhengmai7698 was significantly higher than those in the other wheat cultivars (*p* < 0.05), except for Zhengmai0856 (*p* > 0.05). The Cd concentrations in grains of all the 16 wheat cultivars exceeded the MPC value of 0.1 mg kg^−1^, indicating that there was substantial Cd accumulation in wheat grains in Jiyuan, which agreed with the results observed by Xing et al. [41], who found that Cd concentrations in wheat grains from 25 wheat cultivars were higher than the MPC value. The grain Cd concentration for Luomai26 was the closest to the MPC value (Figure 2b). The Cd concentrations in the grains of Xinong979, AY58, and Zhengmai379 were 0.11, 0.12, and 0.13 mg/kg, respectively, which slightly exceeded the MPC values. In contrast, the concentrations of Cd in grains for Zhengmai7698, Zhengmai0856, and Pingan8 were nearly two to three times higher than the MPC value. However, only four of the 16 wheat cultivars had grain Cd concentrations greater than the European Maximum level set by the Commission Regulation [42], which is 0.2 mg kg^−1^ in the wheat grain. 

The Pb concentrations in the grains ranged between 0.12 mg kg^−1^ in Zhoumai207 and 0.42 mg kg^−1^ in Zhengmai379. The Pb concentrations in the grains were higher than the MPC value for eight wheat cultivars (Bainong58, Zhengmai7698, Zhengmai366, Zhoumai18, Zhengmai379, Zongmai583, and Zhengmai0856). In particular, the concentrations of Pb in grains of Zhengmai366 and Zhengmai379 were two times higher than the MPC value of 0.2 mg kg^−1^. Similar results were found by Liu et al., who observed that the concentrations of Cd and Pb in wheat grains from Zhengzhou were 0.56 and 0.87 mg kg^−1^ [43], respectively, and exceeded the MPC values of 0.1 mg kg^−1^ for Cd and 0.2 mg kg^−1^ for Pb (GB2746-2017) [40].

### 3.4. Correlation of Grain As, Cd, and Pb Concentrations and Soil Properties

Soil properties, such as pH, organic matter, and CEC, can have a remarkable influence on the availability of As, Cd, and Pb in soils, and then affect the variation of heavy metal accumulation in crops [44]. Spearman rank correlation analysis showed the correlations of BCFgs with the soil parameters (Table 4). Soil pH showed a significant negative correlation with wheat grain Cd and Pb (*p* < 0.05) while a positive correlation with As (*p* < 0.05). Similar results were reported by Cheng et al. [45] and Spiegel et al. [46], who found a negative relationship between soil pH and Cd. However, a positive correlation of soil pH and grain Cd and Pb was observed by Hussain et al. [47]. Liu et al. reported that pH was the most important factor contributing to the Cd uptake [48]. As seen in Table 4, As, Cd, and Pb concentrations in wheat grain showed a negative correlation with soil OM. Hussain et al. showed that soil OM was significantly negatively related with wheat Pb, while not being significantly related with wheat Cd [47]. In contrast, Schweizer et al. observed that soil properties did not show correlations with wheat grain Cd concentrations [49]. In addition, chloride concentration and Ca content in the soils can affect the availability of heavy metals [50]. Some chloride forms of Pb or Cd may enter the roots and then enter the cell as Cd or Pb ions [51]. It was worth noting that the chloride salinity may reduce the accumulation of Cd in shoot and roots, and nitrates increased Cd accumulation in the crops [52]. Sarwar et al [53] reported that Ca levels in the soil profile increased Cd availability because it was substituted by Ca, thus increasing absorption by the plant.

### 3.5. Bioaccumulation Factors (BCFs) in Different Wheat Cultivars

**Figure 3:**
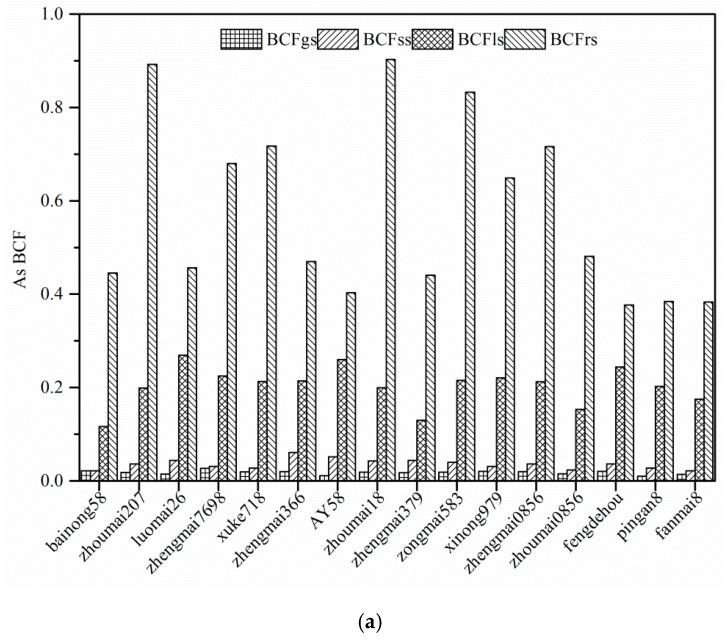

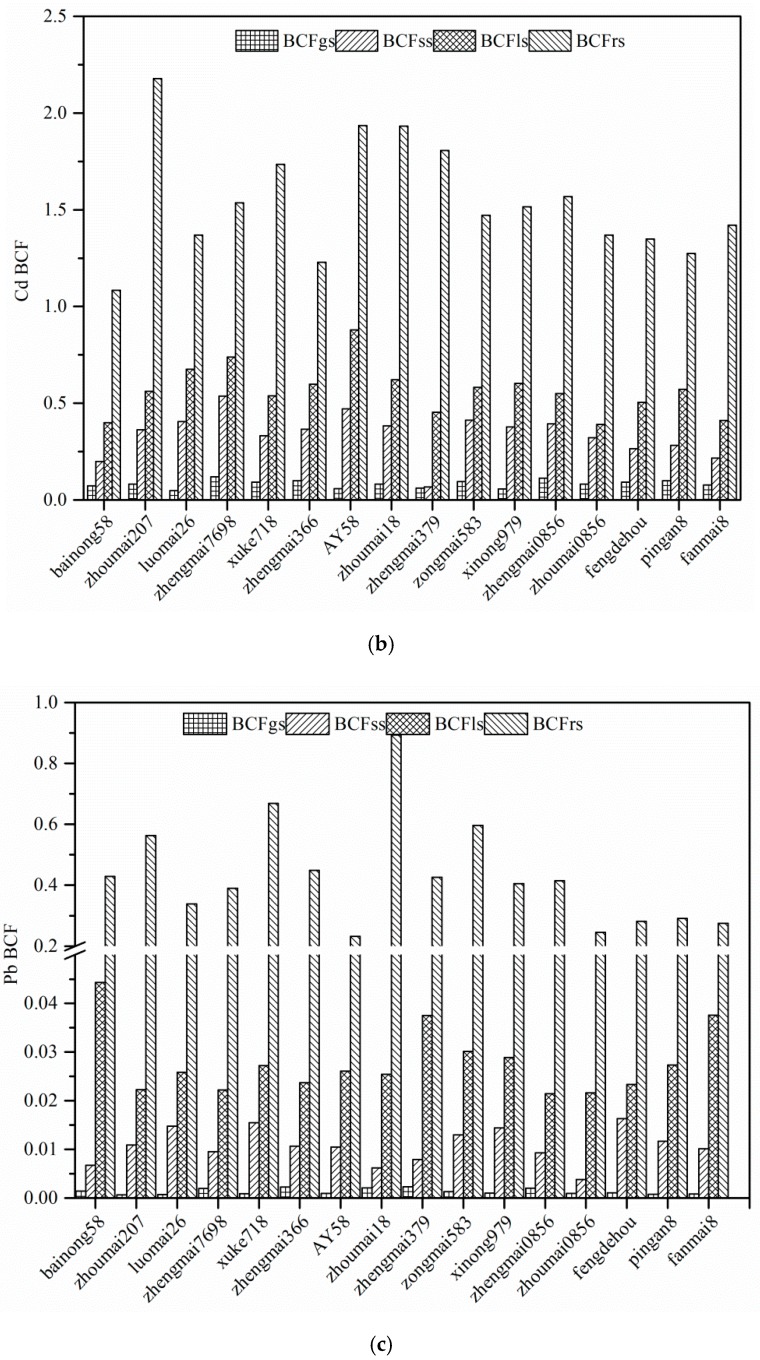
Bioaccumulation factors (BCFs) of As (**a**), Cd (**b**), and Pb (**c**) in different tissues of the 16 wheat cultivars.

BCFs are usually used to evaluate the ability of plants to accumulate As, Cd, and Pb in their tissues. The BCFs of As, Cd, and Pb in different tissues for 16 wheat cultivars are shown in Figure 3. There were obvious differences in the BCFs of As, Cd, and Pb among the 16 wheat cultivars because of cultivar-specific differences, which agrees with Jamali et al. [54], who found that BCFs of heavy metals differed among different wheat cultivars. The BCFs for the 16 wheat cultivars decreased in the order of BCFrs > BCFls > BCFss > BCFgs. On average, the BCFrs values for As, Cd, and Pb were 31.67, 19.5, and 320.8 times higher, respectively, than the BCFgs values. The results agreed with the As, Cd, and Pb concentrations obtained in the tissues of the 16 wheat cultivars, indicating that As, Cd, and Pb were mainly accumulated in the root. These results can be explained by the fact that As, Cd, and Pb were mainly trapped in the roots and transportation from root to shoot was limited [43,55], because As, Cd, and Pb are toxic elements that are not required for crop or vegetable growth.

Wheat species varied widely in their ability to take up and accumulate As, Cd, and Pb, even among cultivars in the same species. The BCFrs, BCFss, BCFls, and BCFgs values of As for the 16 wheat cultivars ranged from 0.37 for Fengdehou to 0.90 for Zhoumai18, 0.021 for Bainong58 to 0.06 for Zhengmai366, 0.116 for Bainong58 to 0.269 for Luomai26, and 0.010 for Pingan8 to 0.027 for Zhengmai7698, respectively. The BCFrs of As in Zhoumai18 was significantly higher than those in other wheat cultivars, except for Zhoumai207 and Zongmai583. The As BCFrs values in this study were higher than those obtained by Liu et al. [43], who found As BCFrs values in the range of 0.29–0.53. However, the As BCFgs values in this study were an order of magnitude higher than those reported by Zhao [56] for wheat (range 0.0004–0.01; mean 0.003) and Williams [57] for wheat (range 0.0001–0.024; mean 0.004). BCFgs of As in pingan8 and AY58 were lower than those in other wheat cultivars, and no significant difference was found between them.

The Pb BCFrs, BCFls, BCFss, and BCFgs values ranged from 0.23–0.89, 0.021–0.044, 0.0038–0.016, and 0.0007–0.0024, respectively, for the 16 wheat cultivars, with all the values below 1.0. Similar Pb BCFrs values were observed for the wheat (0.27–0.43) studied by Liu et al. [43]. Zhoumai18 had the highest Pb BCFrs among the 16 wheat cultivars, while the lowest Pb BCFrs was found in AY58. The highest Pb BCFgs values were observed in Zhengmai366 and Zhengmai379; there was no significant difference between them. The lowest Pb BCFgs values were found in Zhoumai207 and AY58.

The Cd BCFrs values exceeded 1, ranging from 1.08 for Bainong58 to 2.17 for Zhoumai207, implying that the roots of the 16 wheat cultivars had a great Cd accumulation capacity. Stolt et al [58]. demonstrated that the retention of Cd in the root cell vacuoles might influence the symplastic radial Cd transport to the xylem and further transport to the shoot of the plant. However, the Cd BCFss, BCFls, and BCFgs values were below 1, indicating a low accumulation ability of Cd in wheat stems, leaves, and grains. The Cd BCFgs in Luomai26 was lower than those in other wheat cultivars, except for AY58 and Xinong979.

There were substantial differences in the accumulation of As, Cd, and Pb within the same wheat cultivar in this study. The Cd BCFs for all 16 wheat cultivars were higher than those of As and Pb. These results indicated that the ability for Cd accumulation in wheat was higher than that for As and Pb. Similar results were found by Liu et al. [59], who reported that the low uptake and internal distribution of Cd may affect the accumulation difference of Cd in the plants. Relative low BCFgs was found in AY58, which was a potential low accumulation wheat cultivar for the soils contaminated by multiple metals.

### 3.6. Health Risk Assessment of As, Cd, and Pb via Wheat Consumption

The THQ values of As, Cd, and Pb via wheat consumption for children and adults are presented in Table 5. The THQ values of As, Cd, and Pb in this study were higher than those in wheat samples studied by Huang et al. [60], which might be related to the difference in wheat cultivars and environmental conditions. The Pb THQ values in 16 wheat cultivars were the lowest among the heavy metals investigated, ranging from 0.101 to 0.304 for children and from 0.082 to 0.291 for adults. The Pb THQ values for both children and adults were below 1, indicating that daily intake of Pb via wheat consumption would be unlikely to cause adverse human health effects in the study area.

The Cd THQs of 16 wheat cultivars for both children and adults were lower than the safe level of 1, indicating no potential human health effects. The lowest Cd THQ values were found in Luomai26, with values of 0.258 for children and 0.247 for adults. In contrast, the Cd THQ values in Zhengmai7698 were 0.630 and 0.603 for children and adults, respectively, indicating higher health risk when compared with other wheat cultivars. The Cd THQs in this study were comparable to the values found in wheat from Sweden [47]. However, it was noticeable that the health risk of Cd via wheat consumption was negligible despite high Cd concentrations in wheat grains (0.10–0.25 mg kg^−1^ > MPC value of 0.1 mg kg^−1^) (Figure 2b).

The As THQs of 16 wheat cultivars for both children and adults exceeded 1, although the As concentrations in the 16 wheat cultivars were lower than the MPC value of 0.5 mg kg^−1^ (Figure 2a). These results suggested potential human health risks were posed by As via wheat consumption in this study. The As THQ values in Zhengmai7698 were the highest, with values of 2.855 and 2.731 for children and adults, respectively. The As THQ values in Pingan8 were 1.084 and 1.038 for children and adults, respectively; the potential effects of As were lower in Pingan8 when compared with the other wheat cultivars. The potential health risks posed by the individual metals to both children and adults decreased in the order of As > Cd > Pb, indicating As posed stronger potential human health effects than the other heavy metals.

The TTHQ values of 16 wheat cultivars for both children and adults were higher than the safe level of 1, indicating the potential human health risk in the study area. The highest TTHQ values of heavy metals were found in Zhengmai7698, with values of 3.743 and 3.587 for children and adults, respectively. The lowest TTHQ values were found in AY58, with values of 1.677 and 1.605 for children and adults, respectively, suggesting that AY58 posed lower potential detrimental human health effects when compared with the other wheat cultivars tested. These results indicated that AY58 was a very promising cultivar for wheat production in multiple heavy metal contaminated soils to reduce the potential health risk.

The THQ and TTHQ values of As, Cd, and Pb via wheat consumption for children were higher than those for adults, indicating that children were more vulnerable to As, Cd, and Pb than adults in the study area. 

## 4. Conclusions

The investigation in this study showed that the significant differences of As, Cd, and Pb in wheat grains were found among the 16 wheat cultivars, suggesting it was possible to screen the wheat cultivar with a low accumulation ability. Soil pH, OM, and CEC have an influence on the concentrations of As, Cd, and Pb in wheat grains. AY 58 was found to have relatively low grain Cd and Pb concentrations compared with other wheat cultivars. In addition, relatively low BCFgs of As, Cd, and Pb was found for AY58, indicating a low accumulation of As, Cd, and Pb from the soils. 

The THQs of Cd and Pb via wheat consumption were below the safe level of 1, indicating health risks from single heavy metals were not significant. However, THQs of As exceeded 1 for all the 16 wheat cultivars, suggesting that the potential health risk of As to the resident were obvious. It was noticeable that the TTHQ values via wheat consumption exceeded 1, indicating the potential human health risk in the study area.

The results raise attention for enforcing more stringent limitations on industrial emissions in order to protect children from contaminated hazards.

## Figures and Tables

**Table 1:** Physicochemical characteristics of the soils in the field experiment site.

Properties	Min	Max	Mean	SD	Standard (GB 15618-2018)
pH	6.83	7.94	7.56	0.31	—
CEC [cmol/kg(+)]	1.04	9.68	7.62	2.69	—
Organic matter content (OM) (g/kg)	15.5	18.10	16.8	1.01	—
Total N (%)	0.123	0.157	0.139	0.012	—
Total P (%)	0.061	0.084	0.069	0.007	—
Total K (%)	1.78	2.09	1.97	0.09	—
Alkali hydrolysable N (mg/kg)	71.3	122.0	89.05	17.52	—
Rapidly-available P (mg/kg)	8.10	46.8	18.5	12.2	—
Rapidly-available K (mg/kg)	66.5	126.0	91.89	20.94	—
As (mg/kg)	14.57	22.40	18.38	1.19	30
Cd (mg/kg)	1.69	2.35	2.06	0.33	0.3
Pb (mg/kg)	142.63	210.23	177.37	13.20	120
Cr(mg/kg)	75.31	90.47	80.81	3.33	200
Cu(mg/kg)	22.60	30.67	25.42	1.99	100
Ni(mg/kg)	23.79	30.87	26.23	1.80	100
Zn(mg/kg)	76.90	102.88	87.15	6.04	250

Min: Minimum values; Max: Maximum values; SD: Standard deviation; CEC: Cation exchange capacity.

**Table 2:** As, Cd, and Pb concentrations in the grain, stem, leaf, and root of 16 wheat cultivars.

Cultivars	Tissue	As (M ± SD)	Cd (M ± SD)	Pb (M ± SD)
Bainong	grain	0.27 ± 0.015	0.15 ± 0.003	0.26 ± 0.012
	stem	0.22 ± 0.024	0.41 ± 0.012	1.19 ± 0.011
	leaf	1.44 ± 0.013	0.82 ± 0.006	7.87 ± 0.038
	root	5.49 ± 0.208	2.23 ± 0.043	76.10 ± 2.513
Zhoumai207	grain	0.23 ± 0.011	0.17 ± 0.013	0.12 ± 0.016
	stem	0.45 ± 0.004	0.75 ± 0.022	1.94 ± 0.021
	leaf	2.45 ± 0.031	1.16 ± 0.038	3.95 ± 0.087
	root	10.98 ± 2.177	4.49 ± 0.231	99.91 ± 2.65
Luomai26	grain	0.18 ± 0.013	0.10 ± 0.123	0.13 ± 0.012
	stem	0.54 ± 0.001	0.84 ± 0.021	2.62 ± 0.032
	leaf	3.31 ± 0.062	1.39 ± 0.014	4.59 ± 0.028
	root	5.62 ± 0.248	2.82 ± 0.078	60.13 ± 2.154
Zhengmai7698	grain	0.34 ± 0.023	0.25 ± 0.004	0.35 ± 0.002
	stem	0.38 ± 0.014	1.11 ± 0.003	1.70 ± 0.026
	leaf	2.77 ± 0.062	1.52 ± 0.017	3.94 ± 0.011
	root	8.37 ± 1.456	3.17 ± 0.125	69.20 ± 3.548
Xueke718	grain	0.24 ± 0.003	0.19 ± 0.009	0.16 ± 0.015
	stem	0.34 ± 0.023	0.68 ± 0.015	2.75 ± 0.041
	leaf	2.62 ± 0.032	1.11 ± 0.035	4.83 ± 0.102
	root	8.83 ± 1.032	3.57 ± 0.041	68.56 ± 6.554
Zhengmai366	grain	0.25 ± 0.121	0.20 ± 0.006	0.40 ± 0.022
	stem	0.25 ± 0.162	0.75 ± 0.013	1.90 ± 0.005
	leaf	2.64 ± 0.245	1.23 ± 0.201	4.20 ± 0.170
	root	5.79 ± 1.658	2.53 ± 0.514	79.69 ± 6.545
AY58	grain	0.14 ± 0.031	0.12 ± 0.024	0.12 ± 0.011
	stem	0.32 ± 0.005	0.97 ± 0.001	1.86 ± 0.021
	leaf	3.19 ± 0.215	1.81 ± 0.245	4.63 ± 0.332
	root	4.96 ± 0.594	3.99 ± 1.064	41.33 ± 5.215
Zhoumai18	grain	0.23 ± 0.102	0.17 ± 0.044	0.37 ± 0.022
	stem	0.27 ± 0.009	0.79 ± 0.035	1.10 ± 0.004
	leaf	2.46 ± 0.129	1.28 ± 0.045	4.51 ± 0.020
	root	11.12 ± 2.129	3.98 ± 0.278	58.55 ± 12.529
Zhengmai379	grain	0.22 ± 0.011	0.13 ± 0.104	0.42 ± 0.042
	stem	0.12 ± 0.001	0.14 ± 0.004	1.41 ± 0.007
	leaf	1.60 ± 0.229	0.94 ± 0.039	6.65 ± 0.059
	root	5.43 ± 0.208	3.72 ± 0.065	75.49 ± 0.075
Zongmai583	grain	0.23 ± 0.021	0.19 ± 0.004	0.24 ± 0.002
	stem	0.49 ± 0.094	0.85 ± 0.062	2.31 ± 0.028
	leaf	2.65 ± 0.121	1.20 ± 0.211	5.35 ± 0.030
	root	10.25 ± 2.541	3.03 ± 0.101	45.78 ± 6.544
Xinong979	grain	0.26 ± 0.120	0.12 ± 0.006	0.18 ± 0.012
	stem	0.38 ± 0.036	0.78 ± 0.017	2.56 ± 0.084
	leaf	2.72 ± 0.102	1.24 ± 0.021	5.13 ± 0.161
	root	7.99 ± 0.169	3.12 ± 0.452	71.85 ± 7.543
Zhengmai0856	grain	0.25 ± 0.004	0.23 ± 0.002	0.36 ± 0.008
	stem	0.30 ± 0.001	0.81 ± 0.030	1.65 ± 0.076
	leaf	2.62 ± 0.042	1.14 ± 0.061	3.81 ± 0.012
	root	8.82 ± 0.298	3.23 ± 0.592	73.48 ± 6.128
Zhoumai0856	grain	0.19 ± 0.012	0.17 ± 0.014	0.17 ± 0.042
	stem	0.24 ± 0.025	0.66 ± 0.085	0.68 ± 0.303
	leaf	1.89 ± 0.108	0.81 ± 0.085	3.84 ± 0.045
	root	5.92 ± 0.218	2.82 ± 0.288	43.68 ± 6.811
Fengdehou	grain	0.25 ± 0.016	0.19 ± 0.024	0.19 ± 0.032
	stem	0.45 ± 0.001	0.55 ± 0.004	2.90 ± 0.011
	leaf	3.00 ± 0.154	1.04 ± 0.128	4.14 ± 0.146
	root	4.64 ± 0.749	2.78 ± 0.303	50.05 ± 4.135
Pingan8	grain	0.13 ± 0.012	0.21 ± 0.025	0.14 ± 0.018
	stem	0.34 ± 0.057	0.58 ± 0.006	2.07 ± 0.025
	leaf	2.50 ± 0.031	1.18 ± 0.075	4.85 ± 0.058
	root	4.73 ± 1.267	2.63 ± 0.013	51.80 ± 6.231
Fanmai8	grain	0.17 ± 0.062	0.16 ± 0.016	0.15 ± 0.002
	stem	0.27 ± 0.036	0.45 ± 0.047	1.80 ± 0.107
	leaf	2.16 ± 0.208	0.85 ± 0.065	6.66 ± 0.037
	root	4.72 ± 0.103	2.93 ± 0.208	48.79 ± 4.208

M: Mean; SD: Standard deviation.

**Table 3:** Statistics summaries of As, Cd, and Pb in the grain, stem, leaf, and root in wheat cultivars (values followed by different lowercase letters represent significant differences in heavy metal concentrations in the wheat tissue).

Tissues	Parameters	As	Cd	Pb
grain	Min	0.13	0.10	0.12
	Max	0.34	0.25	0.42
	Media	0.28	0.19	0.22
	Mean	0.27cd	0.18d	0.24d
	SD	0.17	0.43	0.11
stem	Min	0.12	0.14	0.68
	Max	0.54	1.11	2.90
	Media	0.33	0.75	1.87
	Mean	0.32c	0.69c	1.90c
	SD	0.10	0.23	0.62
leaf	Min	1.44	0.81	3.81
	Max	3.31	1.81	7.87
	Media	2.62	1.17	4.61
	Mean	2.50b	1.16b	4.93b
	SD	0.52	0.26	1.18
root	Min	4.64	2.23	41.33
	Max	11.12	4.49	99.91
	Media	5.85	3.08	72.68
	Mean	7.10a	3.19a	69.65a
	SD	2.34	0.61	18.98

Min: Minimum values; Max: Maximum values; SD: Standard deviation.

**Table 4:** Spearman rank correlation coefficients among soil parameters, As, Cd, and Pb, in 16 wheat grains.

	OM	CEC	pH	As	Cd	Pb
OM	1	−0.343	−0.612 *	−0.218	−0.276	−0.242
CEC		1	0.445	0.300	0.117	−0.633
pH			1	0.308	−0.528 *	−0.599 *
As				1	0.279	0.503 *
Cd					1	0.294
Pb						1

Significance: * *p* < 0.05. OM: Organic matter, CEC: Cation exchange capacity.

**Table 5:** THQ values of heavy metals via wheat consumption for children and adults.

Wheat Cultivars	Children				Adults			
	THQ_As_	THQ_Cd_	THQ_Pb_	TTHQ	THQ_As_	THQ_Cd_	THQ_Pb_	TTHQ
Bainong58	2.286	0.390	0.187	2.862	2.187	0.373	0.179	2.738
Zhoumai207	1.913	0.430	0.086	2.429	1.830	0.411	0.082	2.323
Luomai26	1.540	0.258	0.096	1.895	1.473	0.247	0.092	1.813
Zhengmai7698	2.855	0.630	0.258	3.743	2.731	0.603	0.247	3.581
Xuke718	2.059	0.483	0.117	2.659	1.970	0.462	0.112	2.544
Zhengmai366	2.118	0.523	0.292	2.933	2.026	0.500	0.280	2.806
AY58	1.184	0.311	0.183	1.677	1.133	0.297	0.175	1.605
Zhoumai18	1.967	0.436	0.270	2.672	1.882	0.417	0.258	2.557
Zhengmai379	1.886	0.326	0.304	2.516	1.804	0.312	0.291	2.407
Zongmai583	1.984	0.504	0.171	2.660	1.898	0.482	0.164	2.545
Xinong979	2.187	0.299	0.131	2.617	2.092	0.286	0.125	2.504
Zhengmai0856	2.114	0.598	0.263	2.975	2.023	0.572	0.252	2.847
Zhoumai0856	1.601	0.430	0.124	2.155	1.532	0.411	0.118	2.062
Fengdehou	2.138	0.490	0.139	2.767	2.046	0.468	0.133	2.647
Pingan8	1.084	0.529	0.101	1.715	1.038	0.506	0.097	1.640
Fanmai8	1.466	0.410	0.108	1.984	1.402	0.392	0.104	1.898
